# Unexpected clozapine levels: examples from in-hospital laboratory and exploratory analysis of non-linear concentration-dose relationship

**DOI:** 10.1192/j.eurpsy.2026.11774

**Published:** 2026-07-31

**Authors:** A. M. Mach, P. Bieńkowski, S. Tyras, A. Wnorowska, M. Radziwoń-Zaleska, M. Wojnar

**Affiliations:** 1 Department of Psychiatry, Medical University of Warsaw, Warsaw, Poland; 2 Department of Psyc, University of Michigan, Ann Arbor, MI 48109, United States

## Abstract

**Introduction:**

Clozapine (CLO) remains the gold standard for the treatment of drug-resistant schizophrenia. It is widely accepted that there is a linear relationship between clozapine dose and blood concentrations, although deviations from this pattern are frequently observed in clinical practice.

**Objectives:**

The aim of this study was to investigate clozapine dose-concentration relationship using a naturalistic dataset, with a particular focus on verifying the linearity assumption and identifying potential breakpoints.

**Methods:**

The study employed Multivariate Adaptive Regression Splines (MARS) to detect non-linear relationships in the prediction of CLO and norclozapine (NCLO) serum levels. The analysis was based on therapeutic drug monitoring (TDM) data from hospitalised patients and incorporated variables such as daily CLO dose, smoking status, age, sex and co-medications.

**Results:**

Both laboratory medicine specialist and psychiatrist identified the same cases of unanticipated changes in CLO concentrations after CLO dose modifications in our dataset. In line with the above, the MARS models showed a partially non-linear relationship between CLO dose and CLO and NCLO concentrations with hinge points of ~550 mg/day for CLO and ~ 225 mg/day for NCLO. CLO dose and smoking were the most important predictors of CLO levels, but the models explained only about 20% of the CLO concentration variability.

**Conclusions:**

Our data suggest that the non-linear relationship between CLO dose and blood concentrations is a real-life clinical problem with the CLO dose of ~ 550 mg/day as a provisional hinge point.

**Disclosure of Interest:**

None Declared

